# Long-Term Consumption of High-Fat Diet in Rats: Effects on Microglial and Astrocytic Morphology and Neuronal Nitric Oxide Synthase Expression

**DOI:** 10.1007/s10571-016-0417-5

**Published:** 2016-08-19

**Authors:** Kinga Gzielo, Michal Kielbinski, Jakub Ploszaj, Krzysztof Janeczko, Stefan P. Gazdzinski, Zuzanna Setkowicz

**Affiliations:** 1 Department of Neuroanatomy, Jagiellonian University, Gronostajowa 9, 30-387 Kraków, Poland; 2 Military Institute of Aviation Medicine, Krasinskiego 54, 01-755 Warsaw, Poland

**Keywords:** High-fat diet, nNOS, Astrocyte, Microglia, Hippocampus, Sholl analysis

## Abstract

Obesity in humans is associated with cognitive decline and elevated risk of neurodegenerative diseases of old age. Variations of high-fat diet are often used to model these effects in animal studies. However, we previously reported improvements in markers of memory and learning, as well as larger hippocampi and higher metabolite concentrations in Wistar rats fed high-fat, high-carbohydrate diet (HFCD, 60 % energy from fat, 28 % from carbohydrates) for 1 year; this diet leads to mild ketonemia (Setkowicz et al. in PLoS One 10:e0139987, 2015). In the present study, we follow up on this cohort to assess glial morphology and expression of markers related to gliosis. Twenty-five male Wistar rats were kept on HFCD and twenty-five on normal chow. At 12 months of age, the animals were sacrificed and processed for immunohistochemical staining for astrocytic (glial fibrillary acidic protein), microglial (Iba1), and neuronal (neuronal nitric oxide synthetase, nNOS) markers in the hippocampus. We have found changes in immunopositive area fraction and cellular complexity, as studied by a simplified Sholl procedure. To our knowledge, this study is the first to apply this methodology to the study of glial cells in HFCD animals. GFAP and Iba1 immunoreactive area fraction in the hippocampi of HFCD-fed rats were decreased, while the mean number of intersections (an indirect measure of cell complexity) was decreased in GFAP-positive astrocytes, but not in Iba1-expressing microglia. At the same time, nNOS expression was lowered after HFCD in both the cortex and the hippocampus.

**Electronic supplementary material:**

The online version of this article (doi:10.1007/s10571-016-0417-5) contains supplementary material, which is available to authorized users.

## Introduction

In 2014, more than 1.9 billion adults, 18 years and older, were overweight. Of these, over 600 million were obese. Once considered a problem only in high-income countries, obesity has reached epidemic proportions also in low- and middle-income countries (WHO fact sheet no. 311, updated January 2015). In the nervous system, obesity has been linked to increased oxidative stress and inflammation, particularly involving astrocytes and microglia. In the end, obesity results in cognitive decline in humans and impairment in different tasks in animals (Granholm et al. 2008; Kanoski and Davidson 2011). Multiple different approaches to modeling obesity in experimental animals exist (Bray et al. 2004). Variants of high fat, or high-fat and high-carbohydrate (HFCD) diet are experimental tools often used to model the effects of energy dense food. Of special interest are the effects of such a diet on hippocampal function, as numerous rodent studies indicate that highly caloric diets impair function and structure of the hippocampus, leading to alterations in hippocampus-dependent long-term spatial memory (Kanoski and Davidson 2011; Stranahan et al. 2008). There is also mounting evidence that increased inflammation, oxidative stress and altered energy metabolism linked to HFCD consumption are all tied to mechanisms of cognitive decline relevant both to Alzheimer’s disease and normal aging (Uranga et al. 2010). On the other hand, the relationship between dietary fat and caloric intake, age, and cognitive decline is still controversial, with some authors finding either no negative or weakly positive effects of high-calory diets (Beilharz et al. 2015; Patten et al. 2013; Setkowicz et al. 2015; Solon-Biet et al. 2015). For this reason, it is important to further investigate the influence of high-fat and high-carbohydrate diets on brain function at different points during lifespan, especially in mature and aging animals.

Inflammatory function, metabolism, and homeostasis in the aging brain are heavily dependent on microglial and astrocytic function. Microglial cells are the major cellular component of immune system in the brain. In normal physiological states, most microglial cells have multiple branched processes, a morphology that lends itself well to their primary function of monitoring the local environment for signs of excitotoxicity, damage, or infection (Wake et al. 2013). In central nervous system inflammation, these processes shorten and retract, until the resting microglia transform into macrophages capable of phagocytosis (Kettenmann et al. 2013). In the process, microglial morphology is altered in several steps, classically classified as “ramified” (resting), “hypertrophic” and “bushy.” Immunohistochemical stains that reveal the whole expanse of these cells, such as Iba1 (Jinno et al. 2007) allow for quantitative assessment and classification of these changes (Soltys et al. 2001). Functionally, activated microglia release proinflammatory cytokines like tumor necrosis factor or interleukins 1B and 6 (Block et al. 2007), which may interfere with crucial hippocampal function (Hein et al. 2010). It has been suggested that inflammation associated with high caloric diets and obesity, which may result in impaired spatial memory and novel object recognition, could be mediated via the microglia (Broadbent et al. 2004; Lu et al. 2011).

Astrocytes perform a plethora of functions in the normal brain. This includes blood–brain barrier maintenance, metabolic and homeostatic support via ion and neurotransmitter uptake and trafficking, as well as active cross-talk at the synapse, which contributes to synaptic plasticity (Kimelberg and Nedergaard 2010). In response to acute or chronic CNS insult, astrocytes become prone to gliosis, manifesting as increased proliferation and altered morphology. Morphological complexity and the number and span of processes are typically increased, with a concomitant upregulation of GFAP staining, which lends itself to quantitative morphological analysis (Kang et al. 2014; Wilhelmsson et al. 2006). Under gliotic conditions, baseline astrocytic functions are disrupted, due to decreased or shifted expression of crucial proteins (aquaporins, connexins, ion channels or neurotransmitter transporters, among others) (Nagelhus et al. 2013). At the same time, gliotic astrocytes may directly contribute to tissue damage via local proinflammatory signaling (Dong and Benveniste 2001).

Similarly to microglial activation, the occurrence of astrogliosis has been reported in response to high-fat diet feeding in rodents (Buckman et al. 2013; Pistell et al. 2010; Thaler and Schwartz 2010). This gliosis can be driven by many signals, including nitric oxide (NO) production. NO is a secondary neurotransmitter synthesized by several cell types, notably neurons, via neuronal nitric oxide synthase (nNOS), which is known to be upregulated under inflammatory conditions. High level of NO may influence synaptic plasticity and induce cell death, leading to neuronal degeneration (Brown and Bal-Price 2003).

In our recently published study (Setkowicz et al. 2015), we used rats fed a high-fat, high-carbohydrate diet (HFCD), providing 60 % of energy from fat and 28 % from carbohydrates. The animals were maintained on either HFCD or normal chow for a year, to study long-term diet-associated effects in the adult brain. During that time, they underwent behavioral memory tests (8-arm radial maze). ^1^H magnetic resonance spectroscopy in combination with magnetic resonance imaging (MRI) was used to determine hippocampal volume and the concentrations of metabolic markers of neuronal viability, such as *N*-acetyl amino acids. These markers are widely used in human studies of aging and neurodegenerative diseases and their concentrations are decreased in elderly humans (Gazdzinski et al. 2010). Surprisingly, we concluded that HFCD feeding improved metabolic markers, increased hippocampal volume, and resulted in overall better memory performance.

In the present study, we used brain sections obtained from the animal cohort analyzed in the previous study, to assess glial activation by immunohistochemical (IHC) staining for astrocytic marker GFAP and microglial protein Iba-1 in the hippocampus. We also studied nNOS-positive cell abundance in the hippocampus and motor cortex (M1 area). Not much is known about the morphology of microglia in mature rats fed a HFCD, so we attempted to quantify and compare the morphology of astrocytes and microglia in animals fed a normal diet or HFCD for 12 months. To our knowledge, this study is the first to use morphological tools, such as simplified Sholl analysis, to study glial cells in the context of HFCD.

## Materials and Methods

### Animals

Animals were treated according to the method described previously (Setkowicz et al. 2015). Briefly, male Wistar rats aged 45–50 days were assigned to two experimental groups: HFCD and control (CTRL). The rats were kept under controlled temperature (21 ± 2 °C) and illumination (12-h light/dark cycle) for 12 months, housed individually to enable the experimenters to assess per-animal food intake. The HFCD group received a energetically rich diet consisting of 36.5 % lard (47 % saturated fats, 49 % mono- and poly unsaturated fats), 36.9 % sucrose, 14.0 % proteins, 4.0 % vitamins, 1 % cellulose, and 3 % starch/dextrin, essential minerals, and trace elements, while the control (CTRL) animals were given standard laboratory chow (4.7 % lard, 37.0 % starch/dextrin, 25.3 % proteins, 4.0 % vitamins, 3.9 % fiber, and 3.2 % ash and essential minerals and trace elements). In terms of energy intake, in HFCD, 61 % of total calories came from fats, 28 % from sugars, and 11 % from proteins. In contrast, the control chow provided 14 % of energy from fats, 51 % from carbohydrates, and 35 % from proteins (Laboofeed, Morawski).

Both groups had very similar caloric intake and their body weights followed a similar, stably increasing trajectory over the time of the experiment, with no significant differences in total body mass, although HFCD-fed rats had moderately increased blood levels of glucose and ketone bodies (see Setkowicz et al. 2015 for detailed results and discussion). Upon post-mortem assessment, HFCD-fed rats had 38 % larger fat deposits than CTRLs, as evaluated by volume of epididymal fat, a known marker of fat deposits in rats (Setkowicz et al. 2015). At the time of sacrifice, blood samples were also taken, and serum interleukin 6 levels were assessed with a rat IDELISA IL-6 ELISA kit (Empire Genomics) according to manufacturer protocol, to determine whether an inflammatory state is present. All procedures involving the use of animals were approved by the Bioethical Commission of the Jagiellonian University in Krakow, Poland, in accordance with international standards.

### Tissue Processing and Staining

At 12 months of age, animals were sacrificed by a lethal dose of pentobarbital and perfused transcardially with 0.9 % NaCl followed by 10 % formalin in 0.1 M phosphate buffer, pH 7.4. Brains were removed, postfixed for several days, and sectioned into 30 µm-thick coronal slices on a vibratome (Leica VT1000S).

Free-floating slices were then stained overnight with primary antibodies against glial fibrillary acidic protein (GFAP; DAKO Z0334, 1:2000), Iba1 (Wako PDN2194, 1:2000), or nNOS (Sigma N7280, 1:1000) in Tris-buffered saline (TBS, 0.05 M Tris (Sigma T-1378) in 0.9 % NaCl, pH 7.6). Cells were visualized using the avidin–biotin reaction (Vectastain ABC kit, Vector).

### Image Analysis

**Fig. 1:**
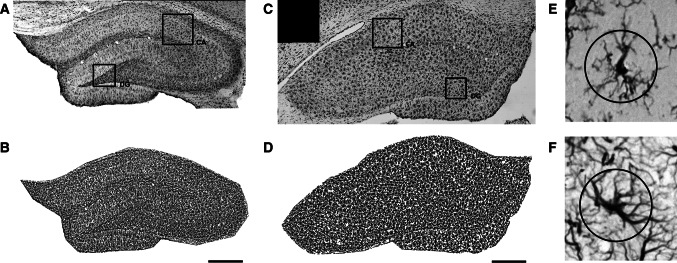
Example representative images of hippocampal staining for GFAP (**a**) and Iba1 (**c**), with corresponding thresholded images (**b** and **d**, respectively). Single cells positive for Iba1 (**e**) and GFAP (**f**) with circular overlays used for cell intersection counting. Areas used for Sholl sampling are indicated on panoramic images (*CA* Ammon’s horn, *DG* dentate gyrus). *Scale bars* 100 µm

Images of stained sections were taken with a digital camera under a microscope (Nikon Microphot SA), with ×10 magnification, and then collected into panoramic images of entire hippocampi with Microsoft ICE software (Fig. 1a, c). Each panorama consisted of 32–42 single frames representing the entire hippocampal formation at Bregma −3.30 to −3.60, according to Paxinos and Watson (1998). The resulting panoramas were analyzed with ImageJ using custom macros for semi-automatic, unsupervised local contrast enhancement and image thresholding (Fig. 1b, d). Briefly, the total area of the hippocampus was outlined manually, the image was split into channels, red and blue channels were discarded, and the green channel was first locally contrast enhanced with the unsupervised CLAHE algorithm implemented in ImageJ (at default settings), followed by automatic local segmentation based on algorithms proposed by Bernsen and Niblack for GFAP and Iba1, respectively. Total GFAP- or Iba1-immunopositive areas in thresholded images were then divided by the area of the entire region of interest encompassing the entirety of the hippocampus to obtain immunopositive area fraction.

In addition to low-magnification panoramic images, a separate subset of images sampled evenly from hippocampal areas CA1 and dentate gyrus (DG) was taken at ×20 magnification. These were then analyzed according to a method described previously (Wilhelmsson et al. 2012). Briefly, single circles of 22 µm diameter—selected to intersect an area with significant process density, encompassing the majority of secondary and some tertiary branches—were overlaid on top of imaged cells, each ring centered on the body of a single cell (Fig. 1e, f). Intersections between the ring and the cell shape were then counted manually, and intersection counts were averaged for all cells in each structure. For Iba1-stained microglia, an average of 54 cells (23–72) were studied per area, while for GFAP-positive astrocytes, an average of 19 cells (12–31) were analyzed. No differences between CA and DG were found (data not shown), and thus, the results were pooled and are reported together here.

For nNOS-positive neuron counts, cells were counted under a ×40 objective, using a 500 μm counting frame overlayed bilaterally on the whole depth of the M1 cortical areas (at around −0.26 to −0.30 from Bregma) and counts from both sides were averaged. Neurons were also counted in hippocampal sections from the same area that was used for glial staining, by examining the whole structure and manually counting visible cells.

### Statistical Analysis

All data are depicted in graphs as full spread with quartiles and median. The data conform sufficiently to assumptions of normality and homoscedasticity (based on visual inspection and *F* ratio testing), and thus, for comparisons between groups, Student’s *T* test was used. All analyses were performed in R statistical software.

## Results

### Area Fraction

**Fig. 2:**
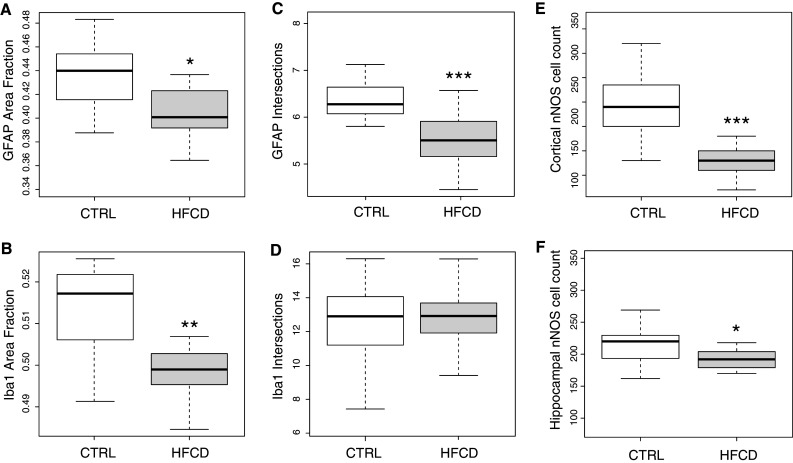
Immunopositive hippocampal area fraction of GFAP (**a**) and Iba1 (**b**). Cell intersection counts at 22 µm from the soma for GFAP-stained hippocampal astrocytes (**c**) and Iba1-stained microglia (**d**). Cortical nNOS-positive cell counts from the M1 area summed bilaterally (**e**). Hippocampal nNOS-positive cell counts obtained from the whole structure (**f**). All graphs represent median (*dark bar*), 25–75 % quartiles (*boxes*) and full span of data points (*whiskers*). *Asterisks* denote significant differences: *p < 0.05; **p < 0.01; ***p < 0.001—Student’s *T* test

Immunopositive area fraction (AF) for GFAP significantly reduced in HFCD-fed animals (mean: 0.402, SD: 0.028, *N:* 15 vs. mean: 0.434, SD: 0.027, *N:* 13 in controls; *p* < 0.005) **(**Fig. 2a**)**. A similar result was observed for Iba1 immunoreactive AF (HFCD mean: 0.499, SD: 0.009, *N:* 16 vs. CTRL mean: 0.512, SD: 0.011, *N:* 19; *p* < 0.003) (Fig. 2b).

### Cell Intersections

For GFAP-positive astrocytes, a significant (*p* < 0.0001) decrease in the number of intersections was found (HFCD mean: 5.5, SD: 0.55, *N:* 24 vs. CTRL mean: 6.4, SD: 0.58, *N:* 16—Fig. 2c). No effect of HFCD on Iba1 cell intersections was detected (*p* > 0.16—Fig. 2d).

### nNOS Cell Counts

In the M1 cortex, there was a marked decrease in nNOS-positive cell count in the HFCD group (HFCD mean: 8.6, SD: 3.99, *N*: 22 vs. CTRL mean: 19.8, SD: 7.50, *N:* 19; *p* < 0.0001—Fig. 2e). A similar but smaller decrease was found in the hippocampal formation (HFCD mean: 186.5, SD: 48.16, *N:* 17 vs. CTRL mean: 218.7, SD: 44.79, *N:* 19; *p* < 0.05—Fig. 2f).

### ELISA

No IL-6 expression was detected in plasma samples from CTRL or HFCD animals at estimated test sensitivity <5 pg/ml.

## Discussion

In our study, we looked for evidence of inflammation or altered glial morphology consistent with the notion of proinflammatory signaling in the brain of obese individuals. In agreement with previous behavioral and metabolic brain imaging results from the same experimental cohort, we failed to find signs of inflammation. A lack of detectable plasma IL-6 suggests that no systemic inflammation was present, although this does not preclude the possibility of local upregulation of proinflammatory signaling, for instance in adipose tissue. In the central nervous system, our approach for identifying microglial and astrocytic activation was based on two parameters: general immunoreactive area fraction, where gliosis is expected to be accompanied by an overall increase in immunoreactivity, and simplified Sholl analysis. In contrast to the original method, described in the classical study (Sholl 1953) and applied to neuronal morphology, simplified Sholl-like methods have been used to rapidly quantify and classify glial cells (Grosche et al. 2013; Sun et al. 2010; Wilhelmsson et al. 2006; Zhang et al. 2012). In this case, cell intersections are counted at one or two predetermined distances from the soma, based on the observation that the difference in process density between activated and resting cells, e.g., astrocytes, tends to be maximized at a distance of 15–25 µm from their center of mass (Kang et al. 2014; Sullivan et al. 2010).

In the present study, we have found a decrease in immunoreactivity for GFAP and Iba1—markers associated with glial activation and inflammation—in the hippocampi of aged animals maintained on HFCD for a long period of time. This corresponds with morphological alterations (decreased number of processes at 22 µm from the soma) and reduced number of nNOS-expressing neurons. It has been previously shown that in reactive astrocytes, the number of intersections a short distance from the soma is elevated compared to nonreactive cells (Wilhelmsson et al. 2006), and increased NO production by nNOS is generally associated with inflammation and neuronal death (Brown and Bal-Price 2003). To corroborate our findings, we performed additional morphometric analyses of single, thresholded astrocytes and microglia, based on previous work (Soltys et al. 2001, 2003, 2005). In these studies, fractal dimension and derivative morphological parameters like form factor, solidity or convexity have been successfully used to discriminate between resting and activated glial cells. Notably, fractal dimension and shape-based metrics (form factor, solidity) were effective at discriminating “ramified” and “hypertrophic” microglial cell types (Soltys et al. 2001). This additional analysis also revealed no signs of gliosis (such as microglial hypertrophy) or increased glial activation in HFCD compared to control animals in any of the parameters studied (Supplementary Material).

Behavioral and metabolic tests performed previously on the same cohort of animals used in this study (Setkowicz et al. 2015) have shown that, in agreement with the results reported here, HFCD consumption improves spatial learning in the radial arm maze, increases hippocampal volume, and results in increased concentration of markers of neuronal viability. Taken together, the behavioral, neuroimaging, and histological data seem to point to an overall improved cognition and lack of central nervous system inflammation in these long-term HFCD-fed rats in comparison with age-matched controls maintained on normal chow.

Calorically rich diet is usually associated with increased inflammation, hippocampal dysfunction, and attenuated learning and memory, both in humans and in animal models (Bray et al. 2004). There are, however, several indications that this relationship is not as simple as it might seem. Exposure to various high-fat diets not always causes neuroinflammation (Boitard et al. 2014; Sobesky et al. 2014). In one particular study (Patten et al. 2013), a net positive effect of diet rich in polyunsaturated fatty acids (PUFA) on hippocampal plasticity and learning was found. Similar studies, showing either no effect or weak positive effect, of high-fat diet on animal performance in hippocampus-associated tasks, water maze learning, and novel object recognition (Beilharz et al. 2015; Pancani et al. 2013) suggest that diets rich in fat and sugar are not necessarily deleterious. A recent study, in which normal chow was compared to low-protein, high-carbohydrate feeding in mice, has shown beneficial metabolic effects (Solon-Biet et al. 2015). Thus, it is possible that our variation of HFCD may have similar effects.

The study most similar to our design was performed by Cano et al. (2014) who fed a high-fat (45 %) diet to mice for 32 weeks. The resulting changes in GFAP-positive cells in the hippocampus were comparable to those seen in our study: a tendency toward cell number decrease, lowered number of astrocyte processes per cell, and increased length of single processes. In a similar study (Patten et al. 2013), the authors, who maintained rats on a PUFA-enriched diet for 11 months, also report moderate positive cognitive effects.

In conclusion, we have observed a surprising, but consistent positive effect of HFCD on hippocampal structure and function in aged rats. This suggests that cognitive decline, which is strongly correlated with obesity on the population level, might not be driven simply by increased fat consumption in and of itself. Taken together with other results that question this simplistic view, we posit that a complex interplay of dietary composition, total caloric intake, the quality and type of fat consumed, as well as interactions with other life-changing phenomena like stress, age, and physical activity should be considered as potential factors influencing the relationship between obesity and cognitive decline. Further studies are required to confirm and expand upon these results, as well as dissect the cellular and molecular mechanisms involved, by quantifying cell numbers and distribution, potential proliferation, and local expression of crucial proteins expressed in glia, such as transporters, chemokine receptors, enzymes (glutamine synthetase), or connexins.

## Electronic supplementary material

Below is the link to the electronic supplementary material.

Supplementary material 1 (XLSX 20 kb)
